# Detection of Isocarbophos Using Porous Silicon Bragg Mirror Combined with Upconversion-Nanoparticle-Based Fluorescence Imaging

**DOI:** 10.3390/bios16100572

**Published:** 2026-10-11

**Authors:** Ziyi Yang, Yangzhi Zhang, Xingyu Wang, Tongzhou Tang, Jiajia Wang, Xiaohui Huang, Zhenhong Jia

**Affiliations:** 1 School of Physical Science and Technology, Xinjiang University, Urumqi 830046, China; yangziyi@stu.xju.edu.cn (Z.Y.); 107552300753@stu.xju.edu.cn (Y.Z.); 107552200733@stu.xju.edu.cn (X.W.); 107552501314@stu.xju.edu.cn (T.T.); 2 Xinjiang Space-Air-Ground Integrated Intelligent Computing Technology Laboratory, Changji 831100, China; 3 School of Information Science and Engineering, Xinjiang University, Urumqi 830046, China; wjjxj@xju.edu.cn (J.W.); hxhdemail@sina.com (X.H.)

**Keywords:** isocarbophos, porous silicon Bragg mirror (PSBM), upconversion nanoparticles (UCNPs), aptamer

## Abstract

To address the difficulty of simultaneously achieving low cost, high sensitivity, and rapid response in the detection of isocarbophos residues in agricultural products and environmental water samples, a portable fluorescence sensing platform integrating a porous silicon Bragg mirror (PSBM) and upconversion nanoparticles (UCNPs) was constructed in this work. First, a PSBM was fabricated by electrochemical etching and then functionalized. Owing to the photonic bandgap effect of the PSBM, which enhances the local optical field, the enhancement effect of the PSBM on upconversion fluorescence was significantly superior to that of single-layer porous silicon. Subsequently, the isocarbophos aptamer was immobilized on the inner walls of the pores of the PSBM, and UCNPs were conjugated with the isocarbophos complementary strand to prepare fluorescent probes. Then, the probe solution was dropped onto the inner walls of the PSBM pores, and through specific binding between the isocarbophos aptamer and the complementary strand, the UCNPs and the complementary strand were indirectly immobilized on the inner walls of the PSBM pores. After that, isocarbophos standard solutions of different concentrations were added. Because isocarbophos molecules have stronger specific binding affinity for the isocarbophos aptamer, the UCNPs and complementary strand originally immobilized on the inner walls of the PSBM pores were displaced by isocarbophos molecules, resulting in a decrease in the number of UCNPs on the inner walls of the PSBM pores and thereby attenuating the upconversion fluorescence. Finally, the surface of the porous silicon sample was irradiated with a 980 nm laser to excite the UCNPs on the inner walls of the pores to generate upconversion fluorescence. Fluorescence images before and after the reaction were recorded by an image acquisition device, and the change in mean grayscale value was calculated using image processing software to determine the isocarbophos concentration. The limit of detection of this method was 0.27 μg/L. Under identical conditions, the signal change caused by 100 μg/L chlorpyrifos, a structurally similar organophosphate pesticide, was negligible, indicating that the sensor has good selectivity for ICP. These results demonstrate that the proposed platform is a promising low-cost, highly sensitive, and rapid tool for pesticide detection. The anti-interference experiment against structurally similar organophosphate pesticides confirmed the selectivity of the aptamer sensing mechanism, while systematic evaluation of matrix effects in real samples remains an important direction for future research.

## 1. Introduction

Isocarbophos (ICP) is an organophosphate insecticide that is widely applied in the protection of grain, oil crops, vegetables, and other agricultural commodities against pests and diseases, and it occupies a central role in agricultural production worldwide. However, ICP exhibits pronounced chemical stability and slow environmental degradation kinetics, leading to persistent residues in non-target environmental matrices [1,2]. Improper application readily causes ICP residues in agricultural products to exceed maximum residue limits (MRLs), after which biomagnification through the food chain introduces the compound into the human body, posing a potential threat to public health [3,4]. Consequently, the development of a sensitive, rapid, and cost-effective method for ICP residue detection is of critical importance for food safety assurance and environmental monitoring.

Current approaches for isocarbophos detection are dominated by chromatographic, fluorescence spectroscopic, and electrochemical techniques. Chromatographic methods, including GC–MS/MS and LC–MS/MS, are internationally recognized and can provide detection limits of 0.1–0.3 μg/kg [5]; however, prohibitively expensive instrumentation and maintenance are required, and the turnaround time for a single sample typically extends to several hours [6]. Nanomolar-level detection limits have been reported for fluorescence spectroscopy [7,8], but the associated instrument costs range from hundreds of thousands to millions of RMB, and each measurement requires 30–60 min. Picomolar-level sensitivity is attainable with electrochemical methods [9], but capital investments ranging from tens of thousands to hundreds of thousands of RMB are similarly required for electrochemical workstations. Collectively, although each of these established techniques excels in one dimension—sensitivity, cost, or speed—the simultaneous integration of all three has not been achieved by any single technique, leaving a persistent gap between analytical performance and practical deployability.

To bridge this gap, a detection strategy combining a porous silicon Bragg mirror (PSBM) with upconversion nanoparticles (UCNPs) was developed in the present study to simultaneously deliver high sensitivity, low cost, and rapid response. UCNPs have emerged as a compelling class of fluorescent sensing materials and have attracted substantial attention recently [10]. They can be excited by 980 nm near-infrared light to emit visible luminescence, and they exhibit weak background fluorescence, excellent photostability, strong resistance to matrix interference, and favorable biocompatibility [11,12,13]. By virtue of these properties, background interference arising from autofluorescence or scattered light in biological and environmental samples is effectively eliminated, while photodamage associated with high-energy ultraviolet–visible excitation is circumvented. Consequently, trace detection in complex matrices is endowed with exceptionally high signal-to-noise ratios and sensitivity. In parallel, porous silicon is characterized by a large specific surface area, good biocompatibility, and facile fabrication into photonic devices [14,15,16]. Compared with our previous work [17], the main advances of this study are as follows: the photonic bandgap effect of the PSBM is exploited to enhance the upconversion fluorescence; an aptamer–cDNA competitive displacement mechanism is introduced for pesticide detection; and selectivity experiments are included to verify the specific recognition of isocarbophos over the structurally analogous pesticide chlorpyrifos. To the best of our knowledge, there is no report combining PSBM with UCNPs for isocarbophos detection. This work systematically evaluates the sensitivity, selectivity, and repeatability of the platform in PBS buffer, validating the sensing mechanism and core analytical performance. The platform offers advantages of low cost, rapid signal readout, and high sensitivity, providing a promising strategy for isocarbophos detection.

The feasibility of competitive displacement of aptamer complementary strands by small-molecule pesticides has been demonstrated in our previous studies on aptamer-based sensors for pesticides and other hazardous substances [18,19,20]. These works provide a solid foundation for the competitive displacement strategy adopted by the PSBM–UCNPs platform in this study.

## 2. Materials and Methods

### 2.1. Fabrication of Porous Silicon

Single-layer porous silicon and porous silicon Bragg mirrors (PSBMs) were fabricated as two types of porous silicon photonic crystals by groove-anode electrochemical etching at room temperature. P-type single-crystalline silicon wafers (〈100〉 orientation, boron-doped, resistivity 0.01–0.05 Ω·cm, thickness 500 ± 25 μm) were purchased from Shandong Yuanjing Electronic Technology Co., Ltd. (Jinan, China) and diced into 2 cm × 2 cm substrates. The etching solution was prepared by mixing anhydrous ethanol and hydrofluoric acid at a volume ratio of 1:1.

#### 2.1.1. Fabrication of Single-Layer Porous Silicon

Single-layer porous silicon was fabricated by electrochemical etching at a constant current density controlled through a LabVIEW interface (National Instruments, Austin, TX, USA). The etching parameters were set as follows: current density, 148 mA/cm^2^; etching duration, 20 s. After etching, the samples were rinsed with deionized water and dried under a nitrogen stream.

#### 2.1.2. Fabrication of Porous Silicon Bragg Mirrors

A porous silicon Bragg mirror (PSBM) is a one-dimensional photonic crystal composed of periodically alternating high- and low-refractive-index layers. It exhibits high reflectivity at the central wavelength within the photonic bandgap and satisfies the Bragg condition [21]:

(1) $$n_{H}\cdot d_{H}=n_{L}\cdot d_{L}=\frac{\lambda_{c}}{4}$$

where $\lambda_{c}$ is the central wavelength of the photonic crystal, $n_{H}$ and $n_{L}$ are the refractive indices of the high- and low-refractive-index layers, respectively, and $d_{H}$ and $d_{L}$ are the corresponding layer thicknesses.

In this study, PSBMs with a 24-layer structure were fabricated, and the wavelength corresponding to the minimum reflectivity at the photonic bandgap edge was set to 650 nm. The etching parameters for the high-refractive-index layers were a current density of 75 mA/cm^2^ and a duration of 1.2 s, whereas those for the low-refractive-index layers were a current density of 148 mA/cm^2^ and a duration of 0.8 s. To ensure a uniform electrolyte concentration, a 3 s interval was applied between the etching of adjacent layers.

**Figure 1:**
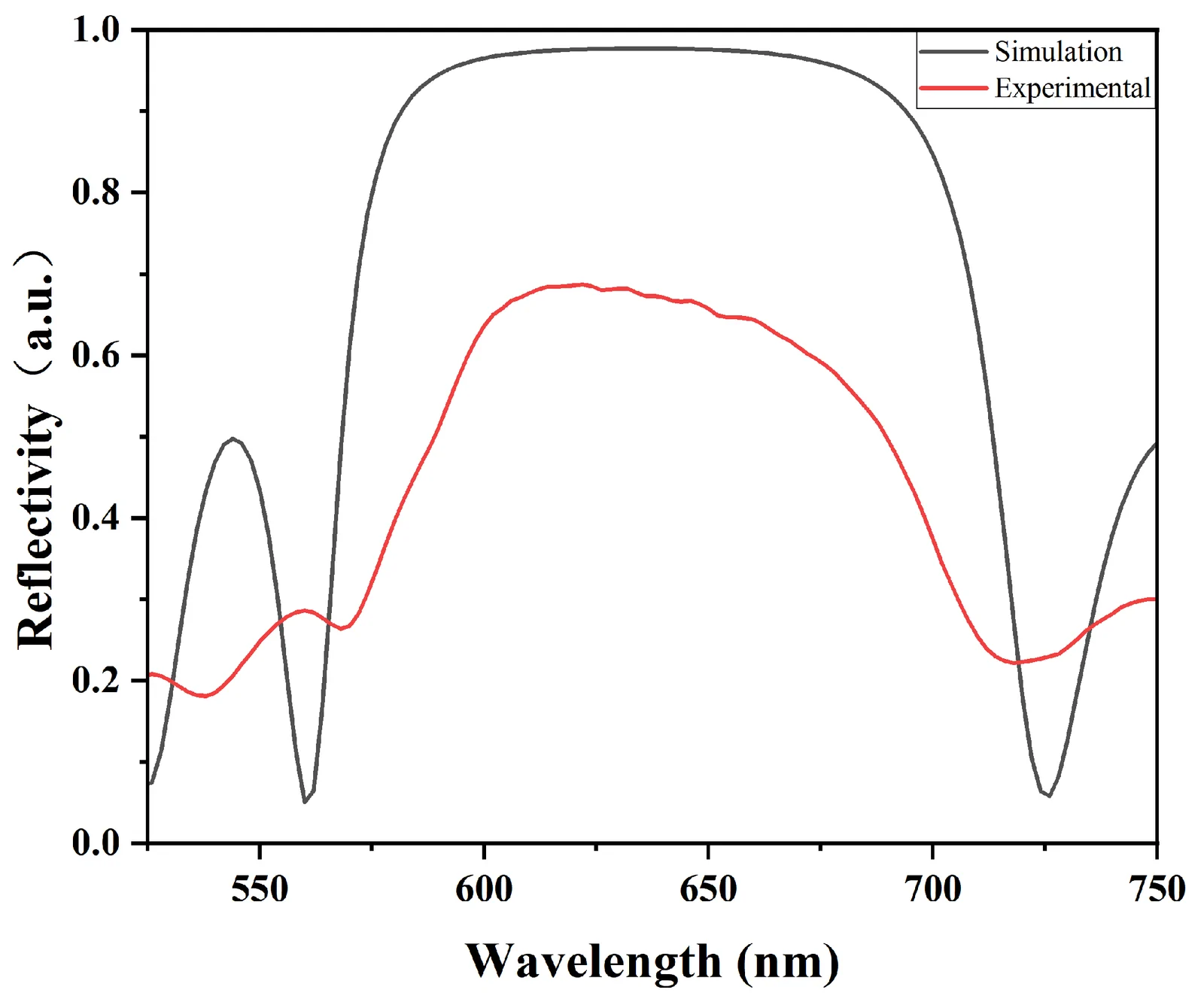
Comparison of experimentally measured and theoretically simulated reflectance spectra of the porous silicon Bragg mirror.

According to the transfer matrix simulation, the periodic parameters of the PSBM were set as follows: the high-refractive-index layer has $n_{H}=1.50$ and $d_{H}=108.3$ nm; the low-refractive-index layer has $n_{L}=1.15$ and $d_{L}=141.3$ nm. The calculated values $n_{H}d_{H}=162.45$ nm and $n_{L}d_{L}=162.5$ nm are both equal to $\lambda_{c}/4=650/4=162.5$ nm, satisfying the Bragg condition in Equation (1). The reflectance spectrum simulated with these parameters shows a central wavelength near 650 nm, consistent with the experimental measurement (Figure 1, confirming the reliability of the PSBM optical design), confirming the reliability of the PSBM optical design.

### 2.2. Functionalization of Porous Silicon Bragg Mirrors

To enable covalent conjugation of biomolecules onto the inner pore walls of the as-etched PSBMs, the freshly prepared samples were subjected to a three-step functionalization protocol consisting of oxidation, silanization, and glutaraldehyde treatment.

**Oxidation:** The freshly etched PSBM samples were immersed in 40% hydrogen peroxide (Chengdu Kelong Chemical Co., Ltd., Chengdu, China) and placed in a vacuum oven at 60 °C for 3 h. The samples were then removed and thoroughly rinsed with deionized water.

**Silanization:** The oxidized samples were immersed in a silanization solution (5% (3-aminopropyl)triethoxysilane (APTES):methanol:deionized water = 1:10:10; APTES and methanol were purchased from Shanghai Aladdin Biochemical Technology Co., Ltd., Shanghai, China) for 1 h, followed by rinsing with deionized water. To form stable amino groups on the pore surfaces, the samples were heated in a vacuum oven at 100 °C for 10 min.

**Glutaraldehyde treatment:** The silanized PSBM samples were immersed in a 2.5% aqueous glutaraldehyde solution (Wuhan Baixin Biochemical Co., Ltd., Wuhan, China) for 1 h. After removal, the samples were washed repeatedly with phosphate-buffered saline (PBS, pH 7.4) to remove excess glutaraldehyde.

### 2.3. Immobilization of the Isocarbophos Aptamer Within PSBM Pores

The isocarbophos aptamer and complementary strand sequences used in this experiment were adopted from those reported in Ref. [22]. Both the aptamer and the complementary strand were commercially synthesized by Sangon Biotech (Shanghai) Co., Ltd., Shanghai, China. The base sequences are as follows:

- Isocarbophos aptamer: 5′-AGCTTGCTGCAGCGATTCTTGATCGCCACAGAGCT-3′-${\mathrm{NH}}_{2}$-$C_{6}$
- Complementary strand: 5′-GATCAAGAATCGCTGCAGCAAGCT-3′-${\mathrm{NH}}_{2}$-$C_{6}$

The isocarbophos aptamer consists of 40 bases with a theoretical length of approximately 13.6 nm, whereas the complementary strand consists of 30 bases with a theoretical length of approximately 10.2 nm. Both dimensions are smaller than the pore diameter of the porous silicon, thereby permitting efficient penetration into the pores for specific binding reactions. A 40 μL aliquot of 10 μM isocarbophos aptamer solution was dispensed onto the surface of the functionalized PSBM samples. The samples were incubated at 37 °C for 2 h to allow covalent immobilization of the aptamer onto the pore walls via an aldehyde–amine reaction. After incubation, the samples were washed repeatedly with PBS and deionized water to remove unbound aptamers. Subsequently, the PSBM samples were immersed in 3 M ethanolamine hydrochloride solution and incubated at 37 °C for approximately 1 h to block unreacted aldehyde groups within the pores. The samples were then rinsed thoroughly with PBS and deionized water and air-dried.

### 2.4. Preparation of the UCNPs–cDNA Fluorescent Probe

**Figure 2:**
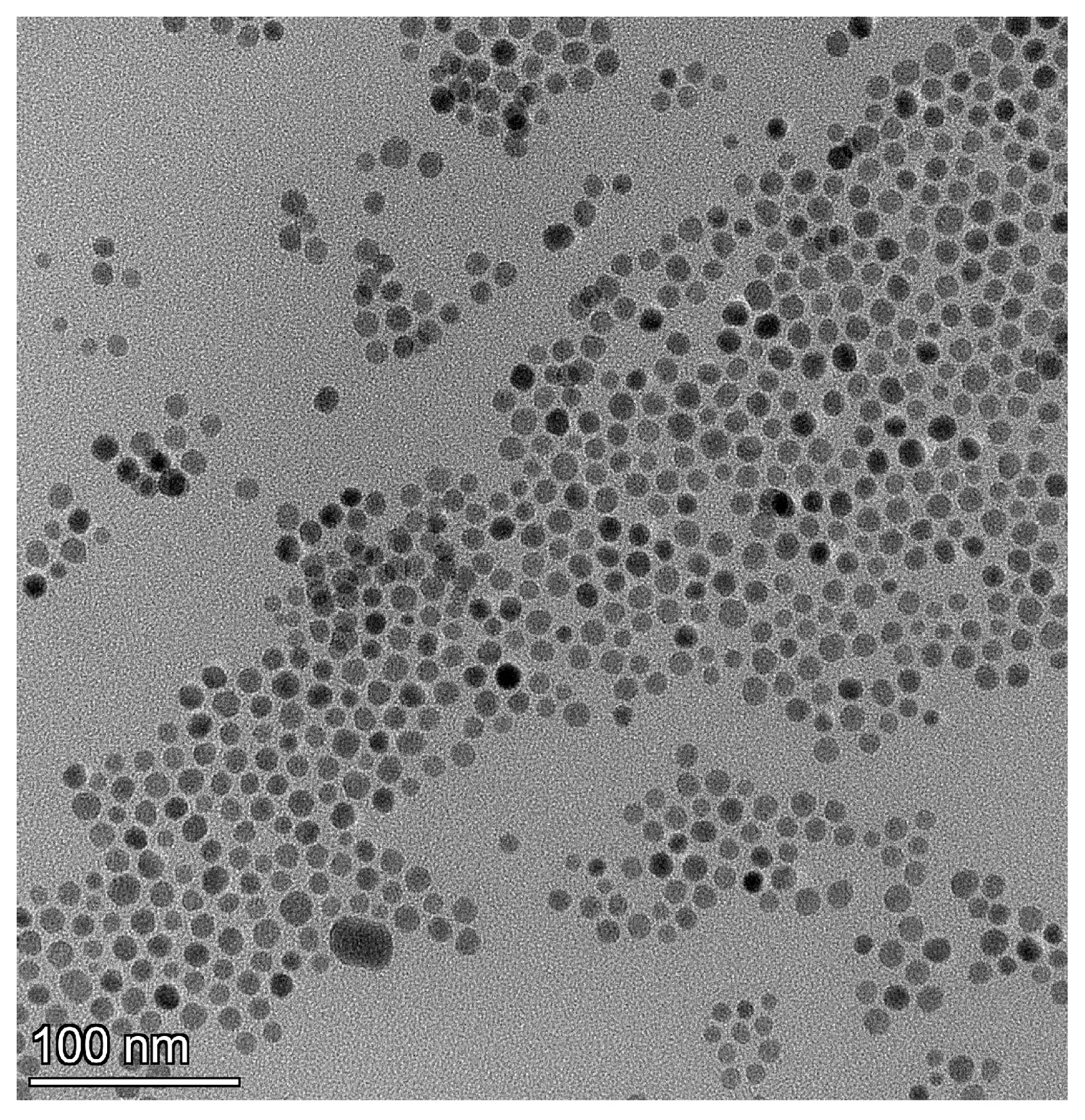
Transmission electron microscopy image of upconversion nanoparticles.

Poly(acrylic acid) (PAA)-modified NaErF_4_:Yb/Tm@NaYF_4_ core–shell upconversion nanoparticles (UCNPs) were used as the fluorescent material in this study and were purchased from Hangzhou Xinqiao Biotechnology Co., Ltd., Hangzhou, China. The nanoparticles were approximately 12 nm in diameter, were excitable by 980 nm near-infrared light, and exhibited a characteristic emission peak at 650 nm. A transmission electron microscopy (TEM) image of the UCNPs is shown in Figure 2.

TEM analysis revealed that the UCNPs exhibit regular morphology and uniform size distribution, with a mean diameter of approximately 12 nm, confirming their suitability for penetration into the porous silicon pores.

The UCNPs-labeled fluorescent probe (UCNPs–cDNA) was prepared strictly following the protocol reported by Zhang et al. [23].

### 2.5. Specific Hybridization of the Isocarbophos Aptamer with UCNPs–cDNA

A 40 μL aliquot of the UCNPs–cDNA probe solution was dispensed onto the inner pore walls of the PSBM pre-immobilized with the isocarbophos aptamer, followed by incubation at 37 °C for 2 h. During this step, hybridization between the aptamer and its complementary strand occurred via Watson–Crick base pairing, forming a stable double-stranded structure. After the reaction, the samples were washed repeatedly with PBS and deionized water to remove unbound UCNPs–cDNA probes, and then dried under a nitrogen stream.

### 2.6. Isocarbophos Detection Experiments

**Figure 3:**
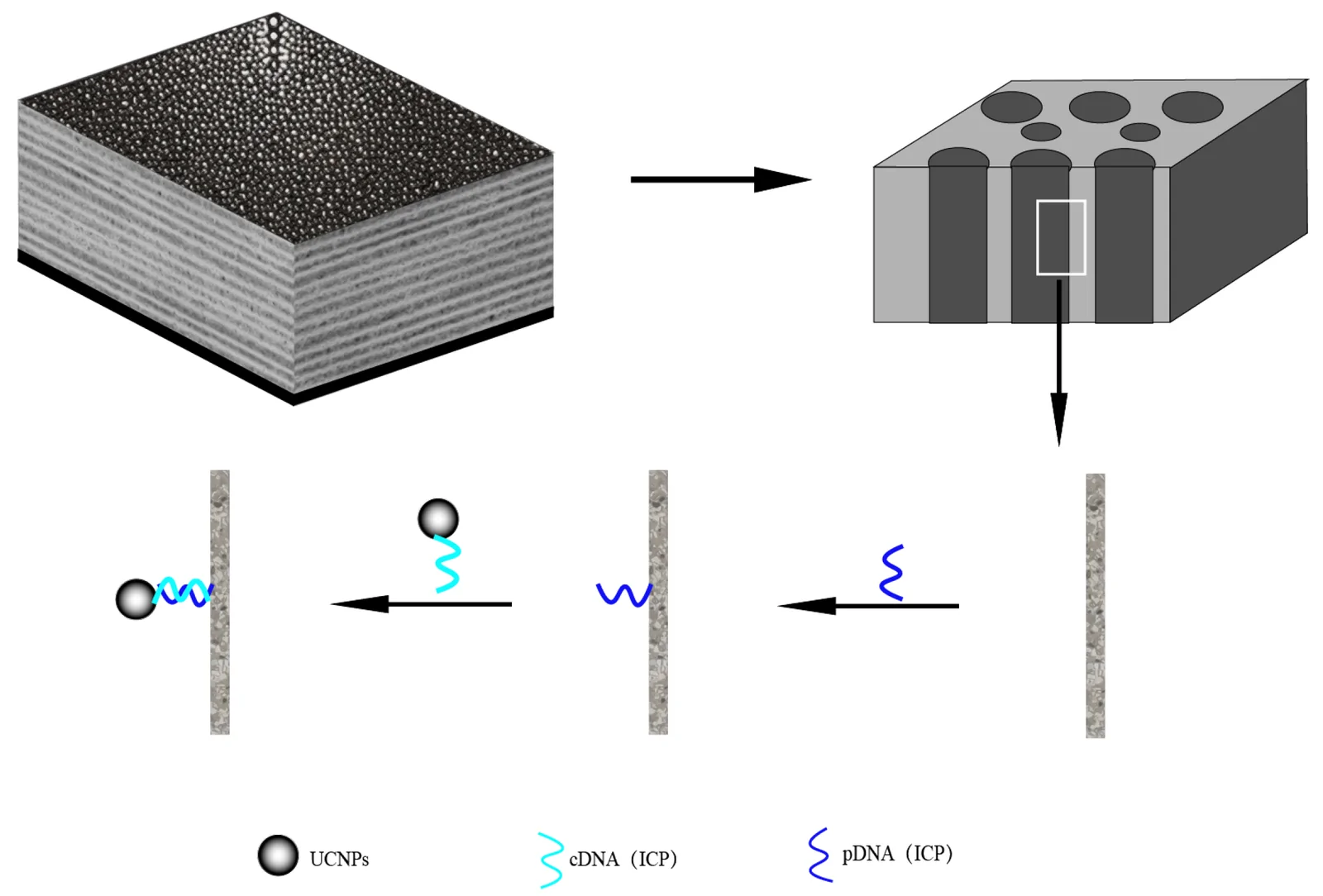
Schematic workflow of isocarbophos detection based on porous silicon Bragg mirrors and upconversion nanoparticle fluorescence imaging.

The isocarbophos standard (molecular formula: $C_{11}H_{16}{\mathrm{NO}}_{4}\mathrm{PS}$; molar mass: 289.29 g/mol) was obtained from Tanmo Quality Control Technology Co., Ltd. (Beijing, China) 0.005 g of isocarbophos powder was dissolved in 2 mL of acetone, ultrasonicated for 5 min, and then diluted to 50 mL with water to prepare a stock solution (100 mg/L). The acetone content in the stock solution was 4% (*v*/*v*). Subsequently, the stock solution was serially diluted with PBS to prepare working solutions of 100, 50, 30, 20, 10, and 5 μg/L. The final acetone concentrations in the working solutions were calculated to be 0.004, 0.002, 0.0012, 0.0008, 0.0004, and 0.0002% (*v*/*v*), respectively. Due to the low water solubility of isocarbophos, acetone was used only for stock solution preparation. After dilution with PBS, the highest working concentration (100 μg/L) is far below its water solubility, and isocarbophos remains dispersed in the aqueous buffer. At these trace levels (≤0.004%, *v*/*v*), acetone does not affect the binding activity of the immobilized aptamer. In addition, all calibration standards were prepared under identical dilution conditions, so any residual solvent effect is systematic. In this work, PBS buffer was selected as the detection medium to provide a controlled ionic strength and pH environment, thereby evaluating the intrinsic analytical performance of the sensor without confounding matrix effects. This is a standard approach in the development of new sensing platforms. For detection, 50 μL aliquots of the working solutions were separately dropped onto the inner pore walls of the PSBM samples with specific binding of the isocarbophos aptamer and UCNPs–cDNA, and incubated at 37 °C for 2 h. For detection, 50 μL aliquots of the working solutions were individually applied to the inner pore walls of the PSBM samples bearing the hybridized aptamer–UCNPs–cDNA complexes, and the samples were incubated at 37 °C for 2 h. Owing to the higher binding affinity of isocarbophos for the aptamer relative to the complementary strand, competitive displacement of the bound UCNPs–cDNA probes occurred. After the reaction, the samples were extensively washed with PBS to remove displaced probes and residual target molecules, dried under a nitrogen stream, and subjected to fluorescence signal acquisition. The complete workflow for fluorescence image-based detection of isocarbophos using the PSBM–UCNPs platform is illustrated in Figure 3.

**Figure 4:**
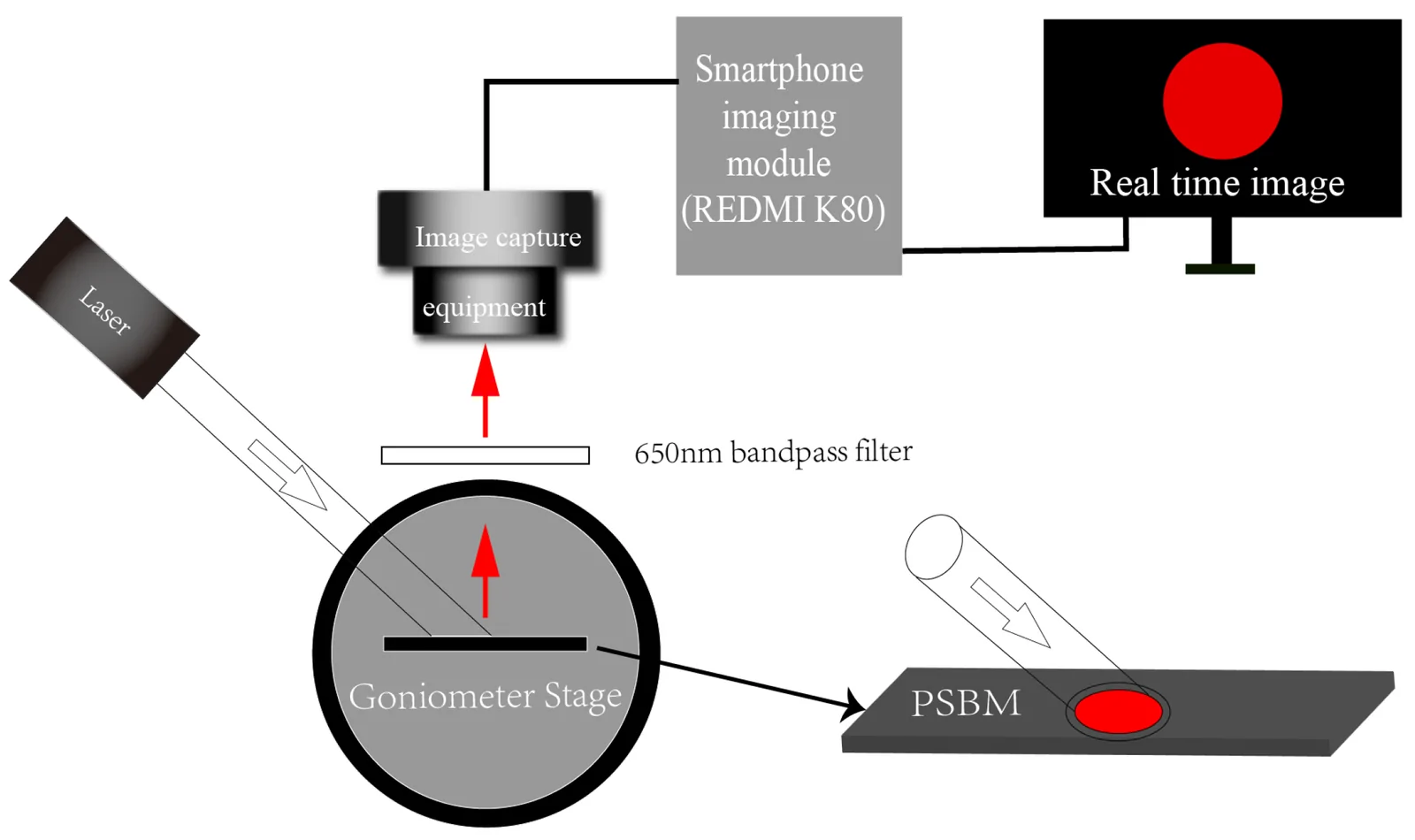
Schematic of the optical path for isocarbophos detection.

Figure 4 illustrates the schematic of the optical path for isocarbophos detection. A 980 nm near-infrared laser (Model MDL-H-980, Changchun New Industries Optoelectronics, Changchun, China) was employed as the excitation source, operating in continuous-wave (CW) multimode mode. The laser beam was directed onto the sample surface at a 45° angle with a source-to-sample distance of 5 cm to excite the UCNPs and generate characteristic fluorescence. After the 980 nm excitation light was filtered out by a BP650 bandpass filter (Shenzhen Nahong Optoelectronics, Shenzhen, China), only the 650 nm upconversion fluorescence was allowed to pass through and was captured by a smartphone (REDMI K80, Xiaomi Communications Co., Ltd., Beijing, China).

All measurements were performed in a dark room with a black light-shielding cover to eliminate ambient light. Fluorescence images were acquired using the same smartphone (REDMI K80), with camera parameters strictly fixed as follows: aperture f/1.6, exposure time 1/14 s, ISO 12800, equivalent focal length 89 mm, and no flash. The smartphone was fixed on a holder at a constant distance of 5 cm from the sample surface and a constant angle of 45°. The excitation source was a 980 nm semiconductor laser with a fixed power of 5 W. Focus and white balance were locked before each measurement. The UCNPs and PSBM samples were stored in the dark at 4 °C before use, and the ambient temperature was maintained at 25 °C during detection. A circular region of interest (ROI) corresponding to 3/4 of the laser spot diameter with uniform grayscale distribution was selected. The mean grayscale values before and after the reaction, $G_{1}$ and $G_{2}$, were calculated, and the grayscale difference $\Delta G=G_{1}-G_{2}$ was used as the quantitative detection signal. The detection of isocarbophos was achieved by establishing a quantitative relationship between isocarbophos concentration and the grayscale difference.

It should be noted that the signal acquisition and processing time in this work is only a few seconds: after incubation and washing, the sample is excited by a 980 nm laser, a fluorescence image is captured by a smartphone, and the image is directly transferred to a computer where the mean grayscale value is calculated by MATLAB R2022b (The MathWorks, Inc., Natick, MA, USA). within seconds. The total assay time, however, includes aptamer immobilization, specific binding of UCNPs–cDNA, target incubation (37 °C, 2 h), washing, and nitrogen drying, giving a total assay time of approximately 2.5–3 h per sample. The target incubation step is necessary for specific aptamer recognition.

### 2.7. Selectivity and Anti-Interference Experiments

Chlorpyrifos standard solution (1000 μg/mL in methanol) was purchased from Tanmo Quality Control Technology Co., Ltd., Beijing, China. A 100 μg/L chlorpyrifos working solution was prepared by two-step dilution with PBS (pH 7.4): 10 μL of the stock solution was diluted to 1 mL with PBS to obtain a 10 μg/mL intermediate solution, and then 10 μL of the intermediate solution was diluted to 1 mL with PBS to obtain the 100 μg/L working solution. An isocarbophos working solution (100 μg/L) was prepared according to the method described in Section 2.6. The PSBM samples with immobilized aptamer and hybridized UCNPs–cDNA probes were divided into two groups: the isocarbophos group (100 μg/L) and the chlorpyrifos group (100 μg/L). A 50 μL aliquot of the corresponding solution was applied to the PSBM surface, followed by incubation at 37 °C for 2 h. After the reaction, the samples were washed repeatedly with PBS and deionized water and dried under a nitrogen stream. Using the same optical system as described in Section 2.6, upconversion fluorescence images were acquired at 650 nm under 980 nm excitation. The mean grayscale values before and after the reaction were recorded, and the grayscale change (ΔG) was calculated. Three replicates were prepared for each group.

## 3. Results and Discussion

### 3.1. Morphology and Characterization of the Porous Silicon Bragg Mirror

**Figure 5:**
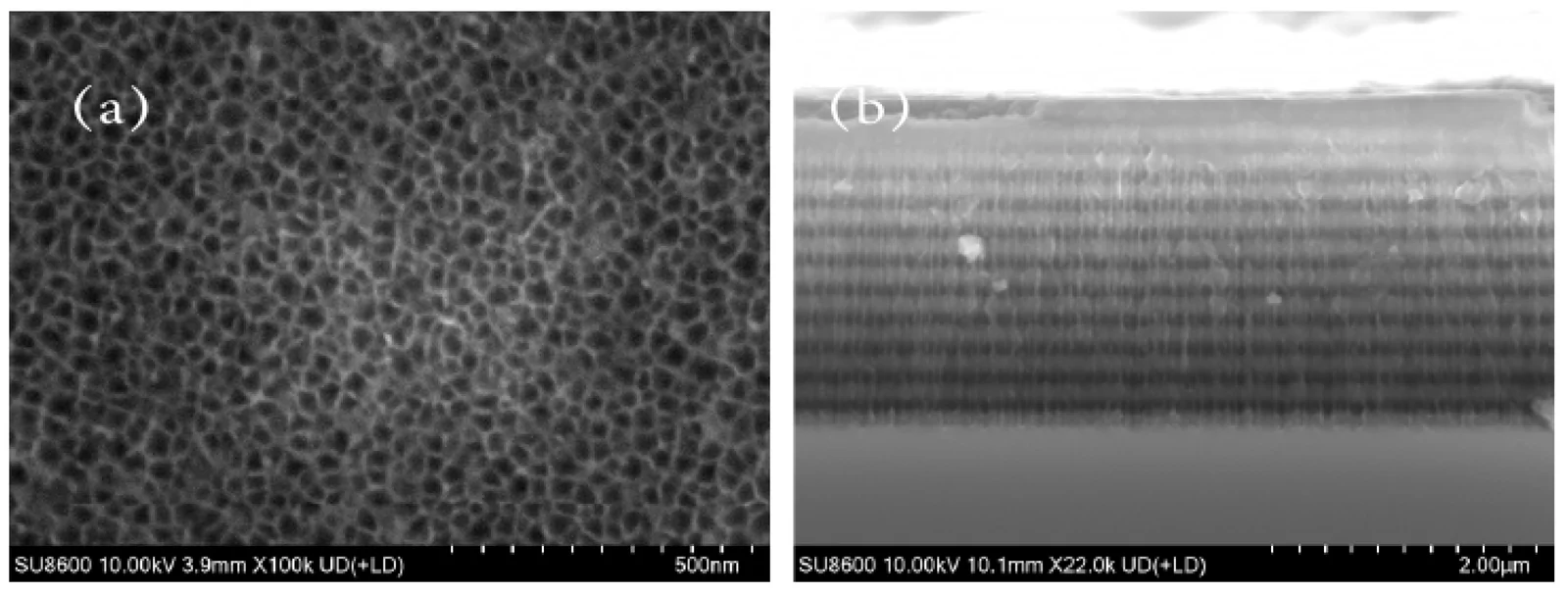
SEM images of the porous silicon Bragg mirror: (**a**) top view; (**b**) cross-sectional view.

As shown in Figure 5a, the as-prepared PSBM exhibits a uniformly distributed surface pore structure with a pore diameter of approximately 50 nm. This pore dimension ensures that the upconversion nanoparticles, isocarbophos molecules, and probe molecules can readily penetrate into the pore channels for specific binding reactions. The cross-sectional image in Figure 5b clearly reveals a well-defined periodic layered structure with uniform thickness across individual layers.

To further validate the optical performance of the PSBM, the reflectance spectrum was theoretically simulated by the transfer matrix method and compared with that experimentally measured from the fabricated device (Figure 1). The simulated and experimentally measured spectra are represented by the black and red curves, respectively. The central wavelength and overall reflectance band profile of the experimental spectrum were found to be in close agreement with the theoretical design, confirming the structural reliability of the fabricated device. The overall reflectance of the experimental curve was slightly lower than that predicted theoretically, which is primarily attributed to scattering and absorption losses of incident light within the porous silicon, as well as additional scattering arising from microscopic roughness at the layer interfaces. Notably, the 980 nm excitation light lies outside the photonic bandgap of the PSBM, which enables efficient penetration into the pore channels for UCNPs excitation. In contrast, the 650 nm upconversion emission generated by the UCNPs under 980 nm excitation falls precisely within the photonic bandgap of the PSBM. Owing to this photonic bandgap effect, the local optical field at the emission wavelength is enhanced within the pore channels, endowing the PSBM with markedly superior upconversion fluorescence enhancement relative to single-layer porous silicon [24]. According to photonic crystal theory, the density of electromagnetic modes within the photonic bandgap is modified, and the interaction between the local optical field and the emitter is enhanced, thereby increasing the upconversion fluorescence intensity. Meanwhile, the high reflectance of the PSBM at 650 nm reflects downward-propagating emission back toward the surface, further improving the forward collection efficiency. It should be noted that the response of a Bragg mirror also depends on the position of the emitter within the standing wave field; in this system, the UCNPs are randomly distributed within the pores, and their positions cannot be precisely controlled, so the degree of local optical field enhancement varies among individual particles. Overall, however, the fluorescence enhancement of the PSBM relative to single-layer porous silicon is attributed to the local optical field enhancement induced by the photonic bandgap effect and the improved emission extraction efficiency [24].

**Figure 6:**
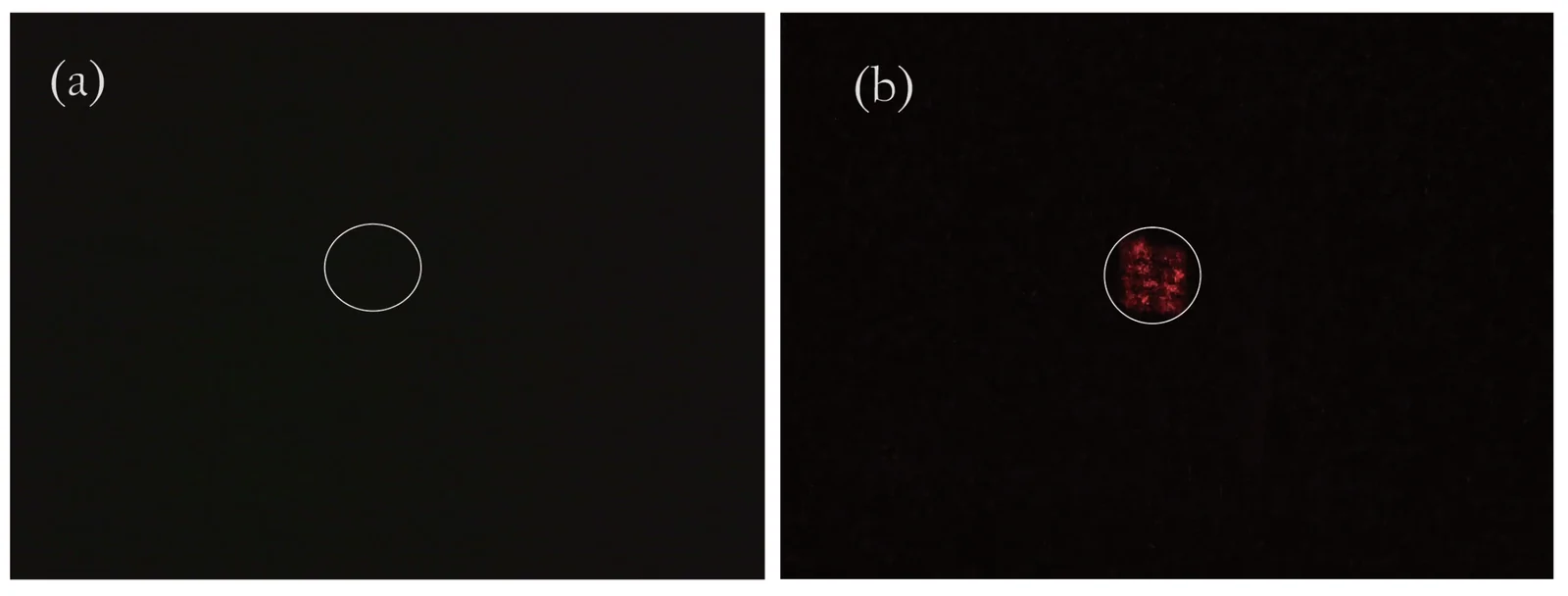
Fluorescence images of porous silicon with immobilized UCNPs: (**a**) single-layer porous silicon; (**b**) porous silicon Bragg mirror.

To verify the fluorescence enhancement effect of the PSBM structure on UCNPs, single-layer porous silicon and PSBM samples were subjected to identical oxidation treatment, after which UCNPs were immobilized on the inner pore walls of both sample types. A 980 nm laser beam, expanded and collimated, was directed onto the samples at a 45° angle. The upconversion fluorescence generated by the UCNPs was filtered through a 650 nm bandpass filter to remove the 980 nm excitation light, and fluorescence images were captured using a smartphone. All experiments were conducted in a dark room to eliminate ambient light interference. The mean grayscale values of the fluorescence images for single-layer porous silicon and PSBM with immobilized UCNPs were determined to be 26.36 and 228.55, respectively. As shown in Figure 6, the fluorescence intensity of the PSBM was significantly higher than that of the single-layer porous silicon, confirming the fluorescence enhancement effect of the Bragg mirror structure.

### 3.2. Reflectance Spectral Shifts During Functionalization

To achieve efficient immobilization of recognition probes on the inner pore walls, the PSBM was subjected to a three-step functionalization protocol consisting of oxidation, silanization, and glutaraldehyde treatment. A change in the refractive index of the medium within the pore channels is induced by each step, thereby causing either a red shift or a blue shift of the PSBM reflectance spectrum. Consequently, monitoring the spectral shift provides a qualitative and intuitive means of assessing the progress of the interfacial chemical reactions.

**Figure 7:**
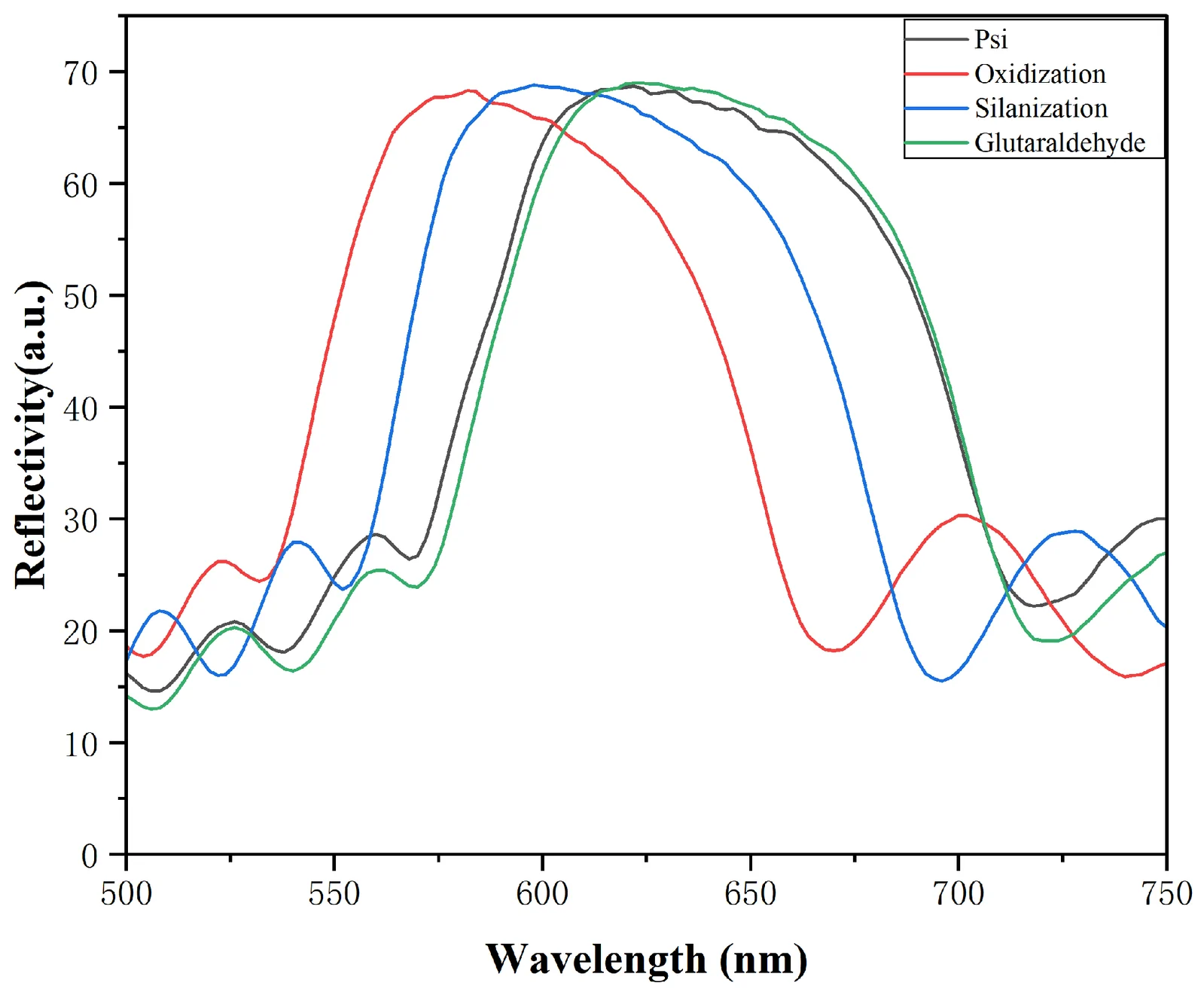
Reflectance spectra of the porous silicon Bragg mirror at each functionalization stage.

The reflectance spectra of the PSBM at each functionalization stage are presented in Figure 7; these spectra were measured using a Hitachi U-4100 UV–Vis–NIR spectrophotometer (Hitachi High-Technologies Corporation, Tokyo, Japan). The black curve corresponds to the pristine reflectance spectrum of the as-etched PSBM, whereas the red, blue, and green curves represent the spectra after oxidation, silanization, and glutaraldehyde treatment, respectively.

The mechanisms underlying the spectral shifts are as follows. During oxidation, a ${\mathrm{SiO}}_{2}$ thin film is formed on the inner pore walls. Because the refractive index of ${\mathrm{SiO}}_{2}$ (≈1.45) is substantially lower than that of crystalline silicon (≈3.4), the effective refractive index decreases, resulting in a blue shift of the reflectance spectrum. Subsequent silanization and glutaraldehyde treatment lead to the stepwise covalent attachment of organic molecules to the pore wall surfaces, thereby partially filling the void volume and increasing the effective refractive index, which manifests as a red shift of the reflectance spectrum. The observed spectral shift patterns are consistent with theoretical expectations, confirming that the functionalization reactions proceeded successfully.

### 3.3. XPS Characterization of UCNPs Attachment and Competitive Displacement

To provide direct chemical evidence for UCNPs attachment and the competitive displacement mechanism, XPS measurements were performed on two groups of samples: (a) PSBM modified with UCNPs–cDNA after functionalization, and (b) PSBM modified with UCNPs–cDNA after further treatment with ICP standard.

**Figure 8:**
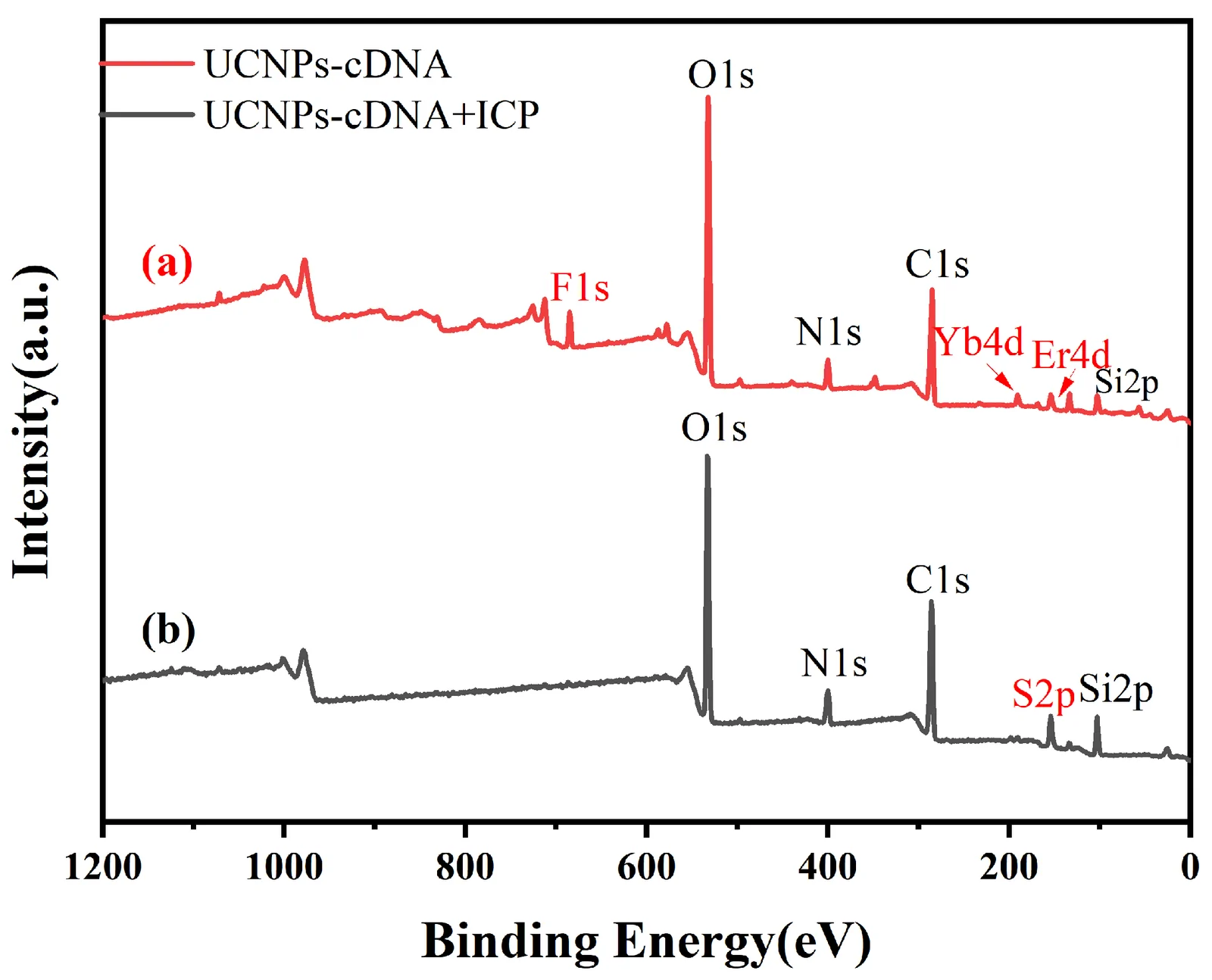
XPS survey spectra of (**a**) PSBM modified with UCNPs–cDNA and (**b**) PSBM after ICP-induced competitive displacement.

As shown in Figure 8, in group (a) (the UCNPs–cDNA curve), C 1s, O 1s, N 1s, and Si 2p signals are observed. Among them, Si 2p and O 1s mainly originate from the porous silicon substrate and its surface oxide layer; C 1s originates from the functionalization layer (APTES and glutaraldehyde) and the DNA strands; and N 1s arises from the DNA bases and the amino groups in the functionalization layer. More importantly, characteristic signals of F 1s, Yb 4d, and Er 4d are clearly observed in group (a). It should be noted that the Yb 4d and Er 4d orbitals of rare-earth elements exhibit spin–orbit splitting (forming 4d_3/2_ and 4d_5/2_ doublets). Because a high pass energy was used for the wide-range survey scan, the resolution is limited, and these split peaks appear as a cluster of adjacent small peaks, resembling a small hump in the spectrum. Despite the complex peak shape, Yb and Er are characteristic constituent elements of the UCNPs (NaErF_4_:Yb/Tm@NaYF_4_), while neither the porous silicon substrate nor the DNA strands contain these elements. Therefore, the appearance of these signals directly confirms that the UCNPs–cDNA probes were successfully attached to the PSBM surface.

After ICP treatment (group b curve), the intensities of Yb 4d and Er 4d decrease markedly compared with group (a), indicating that the UCNPs–cDNA probes were displaced from the PSBM surface. Meanwhile, a new S 2p signal (around 160 eV) appears in group (b). Because neither the DNA strands nor the UCNPs contain sulfur, while the isocarbophos molecule (C_11_H_16_NO_4_PS) contains a P=S group, the emergence of the S 2p signal provides direct chemical evidence that ICP has successfully bound to the aptamer. The above XPS results confirm the successful attachment of UCNPs and the competitive displacement mechanism of ICP on UCNPs–cDNA.

### 3.4. Isocarbophos Detection Results

**Figure 9:**
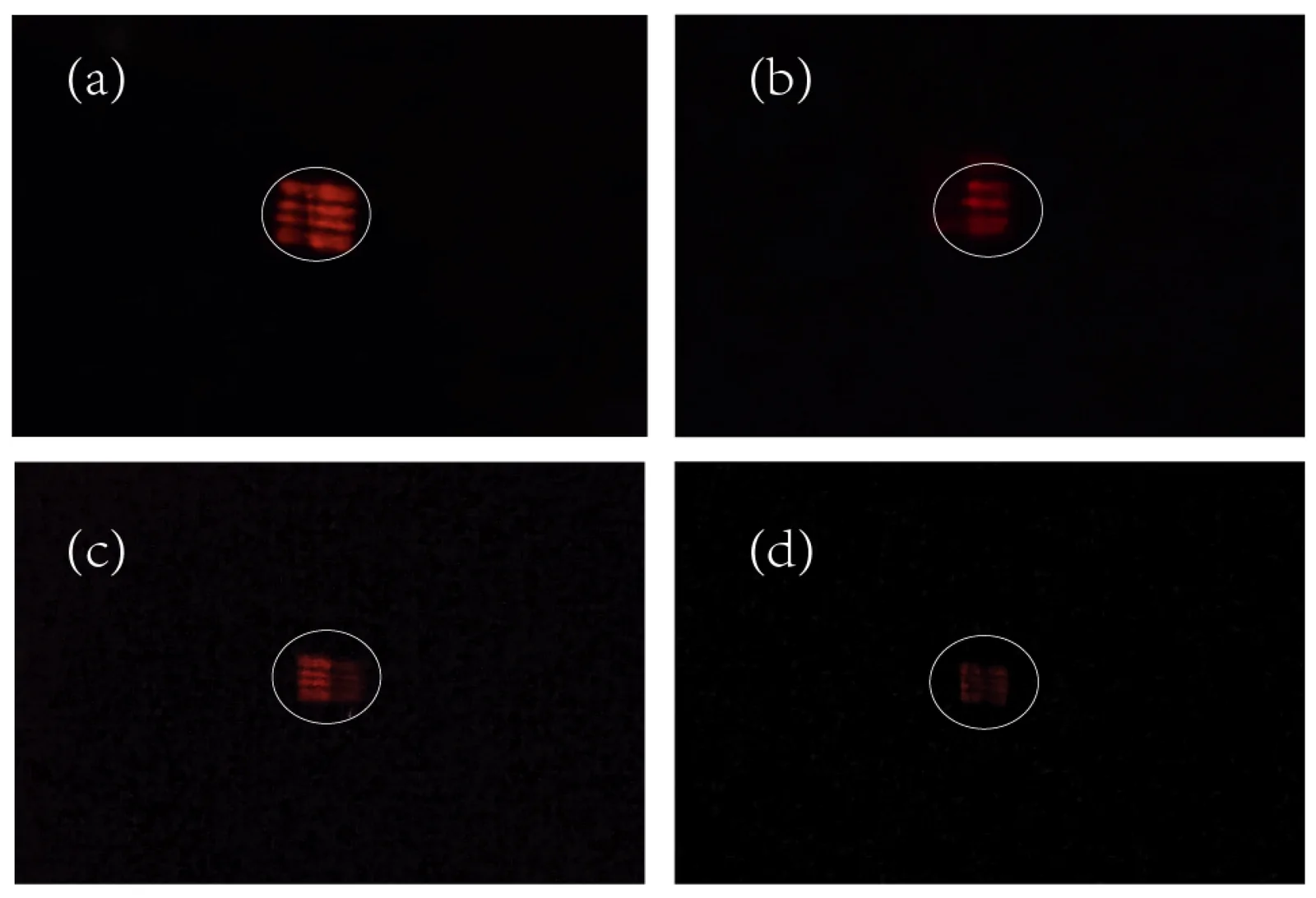
Comparison of fluorescence images before and after treatment with different concentrations of isocarbophos: (**a**) before 100 μg/L isocarbophos; (**b**) after 100 μg/L isocarbophos; (**c**) before 20 μg/L isocarbophos; (**d**) after 20 μg/L isocarbophos.

Figure 9 provides a direct visual comparison of upconversion fluorescence images before and after exposure to different concentrations of isocarbophos. Panels (a) and (c) represent the initial fluorescence images before treatment with 100 μg/L and 20 μg/L isocarbophos, respectively; panels (b) and (d) represent the corresponding fluorescence images after treatment. A pronounced decrease in upconversion fluorescence intensity on the PSBM surface is observed with increasing isocarbophos concentration. This attenuation arises because isocarbophos molecules exhibit stronger binding affinity for the aptamer immobilized on the pore walls than does the complementary strand, displacing the UCNPs-conjugated complementary strands and thereby reducing the number of UCNPs retained within the pores, which in turn weakens the fluorescence signal.

**Figure 10:**
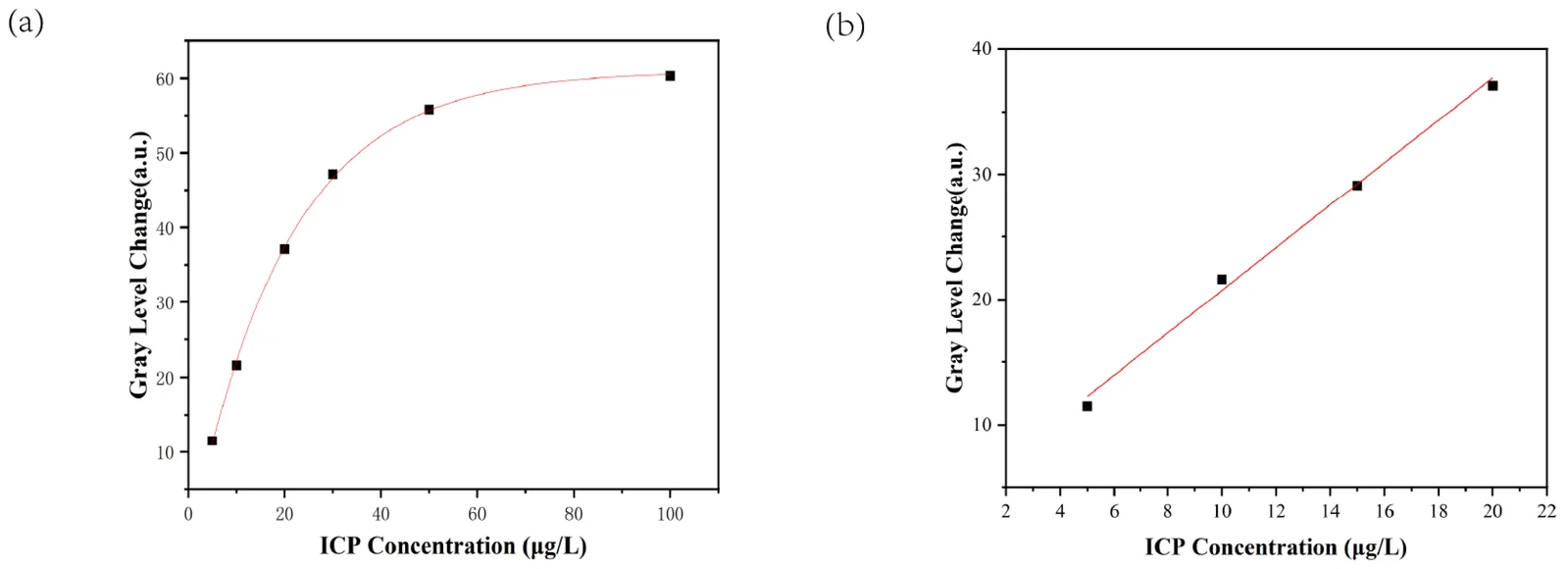
(**a**) Fitted curve of mean grayscale value change versus isocarbophos concentration over 5–100 μg/L. (**b**) Linear calibration curve of mean grayscale value change versus isocarbophos concentration in the low-concentration range (5–20 μg/L).

Figure 10 presents the quantitative calibration curves derived from the fluorescence image analysis. Figure 10a shows the fitted curve of the mean grayscale value change versus isocarbophos concentration over the range of 5–100 μg/L, with the fitting equation:

(2) $$Y=60.96-63.96e^{\left(-\frac{X}{20.01}\right)}$$

where *Y* represents the grayscale change and *X* represents the isocarbophos concentration, with a fitting correlation coefficient of R=0.999.

Figure 10b shows the fitting curve of the mean grayscale value change versus isocarbophos concentration in the low-concentration range. In this range, the mean grayscale change $\Delta G=G_{1}-G_{2}$ exhibits a good linear relationship with isocarbophos concentration, and the linear equation is:

(3) Y=3.75+1.68X

with a linear correlation coefficient of $R^{2}=0.99$, demonstrating that the proposed method enables accurate quantitative detection of isocarbophos.

The competitive displacement mechanism used in this work is supported by our group’s previous studies on aptamer-based sensors for pesticides and other risk substances. In these works, small pesticide molecules were demonstrated to compete with the complementary strand for aptamer binding, leading to displacement of the complementary strand and a corresponding signal change [18,19,20]. These published results, together with the concentration-dependent fluorescence decrease observed in the present study, confirm that ICP can effectively displace UCNPs–cDNA from the aptamer-modified PSBM.

### 3.5. Detection Limit and Linear Range

The inherent topographical undulation of the porous silicon surface can introduce random errors during experiments, potentially affecting detection precision. Therefore, the 3σ criterion [25] was adopted to calculate the method detection limit. The standard deviation was calculated using the formula:

(4) $$\sigma =\sqrt{\frac{\sum_{i=1}^{n}{\left(X_{i}-\bar{X}\right)}^{2}}{n-1}}$$

To accurately evaluate the limit of detection (LOD) of the method, ten independently prepared blank PSBM samples were used in this study. For each sample, ten fluorescence images were acquired under identical conditions, and the average grayscale value of the sample was obtained by averaging the ten images. The standard deviation of the mean grayscale values from the ten independent samples was σ=0.15. This σ includes variations introduced by PSBM fabrication, functionalization, aptamer immobilization, specific binding, and image acquisition, reflecting the overall method uncertainty. According to LOD=3σ/K[25], where *K* is the slope of the linear calibration curve, the LOD was calculated to be 0.27 μg/L. It should be noted that LOD is a statistical detection limit, and being lower than the lowest calibration standard is a common feature of analytical methods, reflecting the high sensitivity of the sensor. The linear range of 5–20 μg/L contains four concentration levels, but each level was measured using ten independent PSBM sensors. The linear fitting was based on 40 independent data points, with $R^{2}=0.99$, confirming that the linear relationship is robust and reliable.

### 3.6. Selectivity and Anti-Interference Performance

To evaluate the selectivity of the proposed sensor, chlorpyrifos, a structurally analogous organophosphate pesticide, was selected as a representative interferent. Under identical conditions, 100 μg/L isocarbophos and 100 μg/L chlorpyrifos were tested. The mean grayscale change (ΔG) for isocarbophos was 61.85±1.46 (mean ± SD, n=3), whereas that for chlorpyrifos was only 1.88±0.93 (n=3). The ΔG induced by isocarbophos was significantly higher than that induced by chlorpyrifos (p<0.05), and the signal change caused by chlorpyrifos was less than 3% of that caused by isocarbophos. These results indicate that chlorpyrifos at 100 μg/L causes negligible signal change and does not interfere with isocarbophos detection, confirming the excellent selectivity of the PSBM–UCNPs sensing platform and the specific recognition capability of the aptamer toward isocarbophos. Since structurally similar organophosphate pesticides are among the most critical interference sources in real environmental and agricultural samples, this anti-interference result supports the potential of the proposed platform for isocarbophos detection in complex real samples. Systematic evaluation of matrix effects in specific real matrices involves sample-specific pretreatment and is a separate research task.

In addition, to evaluate the robustness of smartphone-image-based quantitative analysis, this study minimizes the influence of parameter fluctuations by strictly fixing acquisition parameters, unifying ROI selection, and using the differential signal $\Delta G=G_{1}-G_{2}$. All images were acquired in a dark room using the same smartphone with fixed distance, angle, exposure time, ISO, and laser power (see Section 2.6 for details). The ROI position and size were kept unchanged throughout the experiments, and image subtraction before and after the reaction further cancels fixed background noise. The above standardized procedure and differential strategy ensure that grayscale changes mainly reflect changes in upconversion fluorescence intensity. Although no dedicated repeatability test was performed in this work, this strategy provides a reasonable methodological basis for smartphone-image-based quantitative analysis. Future work will include a systematic repeatability evaluation.

### 3.7. Repeatability and Stability

The stability and repeatability of a sensor are critical parameters for practical deployment. In this study, ten PSBM sensors were fabricated under identical conditions and tested with 100 μg/L isocarbophos. The mean grayscale values of the fluorescence images obtained from the ten sensors gave a relative standard deviation (RSD) of 1.63%. Subsequently, the sensors were measured every five days over a 15-day period, and grayscale changes of 61.68, 61.27, 59.92, and 59.67 were recorded. A slight attenuation of the fluorescence grayscale values was observed over this period, which was tentatively attributed to the detachment of a small fraction of UCNPs or the natural degradation of isocarbophos in the system. Nevertheless, more than 95% of the initial signal was retained, demonstrating excellent long-term stability. Overall, these results demonstrate that the isocarbophos aptamer-based fluorescent sensor developed in this study exhibited outstanding stability and repeatability.

### 3.8. Advantages of the Proposed Sensor and Comparative Evaluation

No additional fluorescence quenchers are required in the proposed method, thereby eliminating a source of chemical interference during signal readout that is inherent to conventional UCNPs–quencher sensing systems. To further evaluate the analytical performance of the proposed method, its detection limit (LOD, 0.93 nM) was systematically compared with those of six previously reported isocarbophos detection methods, with all LOD values converted to nanomolar units (Table 1).

As shown in Table 1, in terms of sensitivity, the proposed method is superior to those reported in Refs. [26,29], and slightly lower than that in Ref. [30]. Although the sensitivity of this method is not as high as those in Refs. [27,28,31], compared with the electrochemical workstation and fluorescence spectroscopy schemes listed in the table, this work has the following three advantages:

1. No large-scale spectroscopic equipment is required, and the detection cost is low. Electrochemical and electrochemiluminescence sensors usually rely on a CHI 660E electrochemical workstation for DPV and EIS measurements, with equipment costs of around $10,000; fluorescence and colorimetric optical sensors generally cost $40,000–$50,000. In contrast, the laser used in this method costs only over $1000.
2. Fast detection. This method requires only two steps: capturing an image of the PSBM surface with a smartphone and calculating the mean grayscale value. The entire process takes less than 1 s. In contrast, the single-sample analysis time for electrochemical and electrochemiluminescence sensors typically exceeds half an hour. Even for fluorescence and colorimetric optical sensors, taking the Hitachi F-4600 fluorescence spectrophotometer used in our laboratory as an example, a single spectral scan (580–680 nm) takes nearly 90 s. More critically, to prevent overheating damage caused by prolonged operation of the light source, the instrument must enter a mandatory cooling cycle after approximately 15 min of continuous operation, which severely limits the continuous detection efficiency of batch samples.
3. High-throughput parallel detection. Our group has previously achieved parallel detection of dual target molecules based on a dual quantum dot/porous silicon optical biosensor [32]. On this basis, the porous silicon Bragg mirror used in this study can be further fabricated into a microarray chip. By immobilizing specific probe DNA for various pesticides or pollutants in different pore regions, parallel detection of multiple targets on a single chip can be realized, effectively overcoming the inherent limitations of conventional homogeneous electrochemical sensing systems in multi-target parallel analysis.

In summary, this study combines a porous silicon Bragg mirror with upconversion nanoparticles and proposes an isocarbophos detection method that integrates high sensitivity, low cost, and rapid response.

## 4. Conclusions

In this study, a porous silicon Bragg mirror (PSBM) with a pore diameter of 50 nm was fabricated by anodic electrochemical etching and subsequently functionalized for biosensing applications. An isocarbophos aptamer was immobilized on the inner pore walls of the PSBM, and upconversion nanoparticles (UCNPs) conjugated to the complementary strand served as fluorescent probes, which were anchored to the pore walls via specific hybridization between the aptamer and the complementary strand. For detection, a 980 nm laser was employed to excite the UCNPs, thereby generating 650 nm upconversion fluorescence. Fluorescence images were captured before and after the competitive displacement reaction, and the change in mean grayscale value was quantified using image processing software. A quantitative relationship between isocarbophos concentration and grayscale change was established, enabling accurate determination of the target analyte. A detection limit of 0.27 μg/L was achieved, with rapid signal readout, low cost, and good sensitivity. Under identical conditions, 100 μg/L chlorpyrifos caused negligible signal change, indicating good selectivity toward isocarbophos. It should be noted that PBS buffer was used as the detection medium in this work to provide controlled ionic strength and pH conditions for establishing and validating the sensing mechanism and analytical performance. The anti-interference experiment against a structurally similar organophosphate confirms the selectivity of the sensor and supports its potential for real-sample analysis. Systematic evaluation of matrix effects in specific real matrices involves sample-specific pretreatment and is a separate research task. Overall, the PSBM–UCNPs platform provides a promising strategy for the rapid and sensitive detection of isocarbophos.

## Figures and Tables

**Table 1:** Comparison of detection limits among different isocarbophos detection methods.

Method	LOD/Sensitivity	Reference
COOH-Apt@UiO-66-NH_2_/AuE electrochemical biosensor	3.11 nM	[26]
Photoinduced regenerative electrochemical aptasensor	3 pM	[27]
PT-ECL hydrogel-based “turn-off” assay platform	20 pM	[28]
β-CDP-based fluorescence enhancement method	4.15 nM	[29]
Dual-mode fluorescence/colorimetric aptasensor	0.207 nM	[30]
Dual-mode immunosensor	0.726 pM	[31]
Our method	0.931 nM	This work

## Data Availability

The data supporting the findings of this study are available from the corresponding author upon reasonable request.
